# Effect of Intermittent Hypobaric Hypoxia Exposure on HIF-1α, VEGF, and Angiogenesis in the Healing Process of Post-Tooth Extraction Sockets in Rats

**DOI:** 10.1055/s-0043-1768639

**Published:** 2023-06-09

**Authors:** Linawati Linawati, Suhardjo Sitam, Wawan Mulyawan, Ambrosius Purba, Achmad Syawqie, Ekowati Handharyani, Yuli Subiakto, Amaliya Amaliya

**Affiliations:** 1 Doctoral Degree Study Program in Military Dentistry Science, Dental Faculty, Universitas Padjadjaran, Bandung, Indonesia; 2 Department of Radiology, Dental Faculty, Universitas Padjadjaran, Bandung, Indonesia; 3 Department of Community Medicine, Universitas Indonesia, Jakarta, Indonesia; 4 Division of Physiology, Department of Biomedical Sciences, Faculty of Medicine, Universitas Padjadjaran, Jatinangor, Indonesia; 5 Department of Oral Biology, Dental Faculty, Universitas Padjadjaran, Bandung, Indonesia; 6 Department of Veterinary Clinic Reproduction and Pathology, School of Veterinary Medicine and Biomedical Sciences, IPB University, Bogor, Indonesia; 7 Military Pharmacy Faculty, Universitas Pertahanan, Jakarta, Indonesia; 8 Departement of Periodontology, Dental Faculty, Universitas Padjadjaran, Bandung, Indonesia; 9 Centre for Military Dentistry Research, Dental Faculty, Universitas Padjadjaran, Bandung, Indonesia

**Keywords:** hypoxia, HIF-1α, VEGF-a, tooth extraction, wound healing

## Abstract

**Objective** The aim of this study was to investigate the effect of intermittent hypobaric hypoxia (IHH) exposure on the expression of hypoxia-induced factor-1α (HIF-1α) messenger RNA (mRNA), vascular endothelial growth factor-a (VEGF-a) mRNA, and angiogenesis after tooth extraction in rats.

**Materials and Methods** On 45 male Sprague-Dawley rats were performed the removal of the maxillary left first molar, and then they were randomly divided into 9 groups, namely: 4 groups that were exposed to IHH for 30 minutes every day in the Hypobaric Chamber at an altitude of 18,000 feet, with 1 time hypobaric hypoxia (HH), 3 times HH, 5 times HH, and 7 times HH; 4 normoxia groups that were terminated on days 1, 3, 5, and 7 after tooth extraction; and the 1 control group. Real-time polymerase chain reaction measured the molecular changes in the socket tissue after tooth extraction in rats to evaluate the expression of HIF-1α mRNA and VEGF mRNA. Histological changes with hematoxylin and eosin staining were noted to evaluate the amount of angiogenesis in the socket after tooth extraction. Molecular and histological parameters were calculated at the end of each experiment on days 0, 1, 3, 5, and 7 after tooth extraction, which exhibited the improvement phase of the wound-healing process.

**Results** Increases in the expression of HIF-1α mRNA, VEGF mRNA, and angiogenesis were found in the IHH group compared with the normoxia group and the control group. The expression of HIF-1α mRNA increased significantly ( *p* < 0.05) in the group after one time HH exposure on day 1, then decreased in the IHH group (three times HH exposure, five times HH exposure, and seven times HH exposure) approaching the control group. The expression of VEGF mRNA and angiogenesis began to increase after one time HH exposure on day 1, and increased again after three times HH exposure on day 3, then increased even more after five times HH exposure on day 5, and increased very significantly (** *p* < 0.05) after seven times HH exposure on day 7. It showed that repeated or intermittent exposure to HH conditions induced a protective response that made cells adapt under hypoxia conditions.

**Conclusion** IHH exposure accelerates the socket healing of post-tooth extraction, which is proven by changes in HIF-1α mRNA expression and increase in VEGF mRNA expression as stimuli for angiogenesis in post-tooth extraction sockets under hypobaric hypoxic condition, which also stimulates the formation of new blood vessels, thereby increasing blood supply and accelerating wound healing.

## Introduction

After tooth extraction, immediately the empty socket is filled with blood resulting from the hemostatic reaction in the alveolar socket, the dynamic interaction of platelets and collagen connective tissue, as well as the balance between coagulation and fibrinolysis, giving rise to the formation of stable blood clots embedded in the fibrin tissue. 1 2 3 4 The wound-healing process after tooth extraction is based on time, following the same pattern as the wound-healing process in general with the inclusion of the socket- and bone-healing process. 1

A few minutes after the tooth is extracted, the blood vessels will experience vasoconstriction as a result of platelet aggregation, causing disruption of oxygen delivery resulting in tissue hypoxia, increased glycolysis, and a decrease in pH, which will be responded by vasodilation; then migration of leukocytes and platelets to the wound tissue occurs. 1 2 Tissue hypoxia serves as a signal that stimulates many aspects of the wound-healing process. 3 5

Hypoxia is a state of reduced oxygen supply to the cellular level that is insufficient to maintain cellular function. 6 Hypoxia can cause platelets and monocytes to release cytokines and growth factors that affect wound healing-cells. 3 5

Changes in oxygen concentration due to hypoxia will modulate cell function by stabilizing hypoxia-induced factor-1α (HIF-1α), which is a transcription factor for many genes that regulate adaptive responses to hypoxia. 7 8 Stabilization of HIF-1α as the main regulator of oxygen homeostasis and determinant of wound-healing outcomes occurs through activation of several HIF-1α target genes. 7 HIF-1α target genes such as vascular endothelial growth factor (VEGF) are significantly elevated in vascular smooth muscle cells that also play a role in angiogenesis, erythropoiesis, energy and glucose metabolism to restore oxygen, nutrient delivery to the wound site, and improving cell viability that promotes wound healing. 7 8

Hypoxia can occur in an environment at an altitude with low atmospheric pressure, where the partial oxygen pressure decreases rapidly as altitude increases, which is known as hypobaric hypoxia (HH). 9 Hypobaric is a condition of environmental changes that occur during the rise in altitude, including changes in air pressure, temperature, and oxygen supply. Correlations between altitude and atmospheric pressure exist. The higher the altitude, the lower the atmospheric pressure or the lower the partial pressure of oxygen, which becomes a stressful condition and affects gas exchange at the cellular level. Cells unresponsive to this stressful condition become alarmingly dysfunctional. 10 The cellular response to HH is complex and characterized by altered expression of several genes including proteins to maintain hemostasis. 11

Some theories explain that the accepted hypobaric hypoxic effect begins to manifest at an altitude of 10,000 feet (3,048 m); at this height the effect of hypoxia on the human body is clearly visible and easily recognizable. 9 12 13 HH during air travel induces several physiological reactions in the human body with changes in gene expression, including related proteins required to maintain homeostasis. Flying for 30 minutes resulted in decreased oxygen saturation and the expression of 10 proteins changed significantly, although the short and moderate HH of protein expression analysis showed its relationship with immune response, protein metabolism, and hemostasis. 14

Hypoxia does not necessarily cause damage to cells and does not necessarily have a negative impact; rather the exposure of mild hypoxia with tolerable levels and periods provides a protective effect (preconditioning), improving adaptive and protective responses so that injuries from subsequent exposure to harmful stimuli are reduced. 15 16 Hypoxia preconditioning has better therapeutic ability than normoxic conditions, which contributes to cell migration and cell survival, and can induce cell repair processes. 15

Adaptation of HH with intermittent hypobaric hypoxic efforts, as part of hypoxia preconditioning, performed with periods of hypoxic exposure interspersed with normoxia repeatedly over a period has several effects on tolerance to subsequent hypoxic exposure, which affects preventing cell damage by reducing oxidative stress and inhibiting the cascade of apoptosis. 16

Pilots and air force flight crews routinely conduct HH training to identify hypoxic conditions and make their bodies adapt to hypoxia. They may experience intermittent hypobaric hypoxia (IHH) in both training and assignment.

Several studies suggest that after IHH exposure at various pressures with an interval of 1 week, with four times of HH induction, there is an increase in HIF-1a of heart and liver tissue after one- and two-time HH induction but continues to decrease back to normal levels after induction of IHH three and four times, meaning that HIF-1a is synthesized only as necessary according to the needs of liver tissue and the heart, which then adapt to conditions of IHH. 17 18 To the best of our knowledge, no studies have been conducted to date to analyze the effect of exposure to IHH on post-tooth extraction socket healing. How the post-extraction molecular and histological changes occur, especially HIF-1α, VEGF, and angiogenesis, in the post-extraction socket after intermittent hypobaric hypoxic exposure remains unclear. Therefore, this study analyzed the molecular and histological changes in HIF-1α, VEGF, and angiogenesis after IHH exposure and its effect on socket healing after tooth extraction.

## Materials and Methods

### Study Design

This research is a true experimental study with a randomized post-test that only controlled group design, using animal models of healthy adult male Sprague-Dawley rats, which was conducted at the Integrated Research Laboratory of the Faculty of Dentistry, Padjadjaran University, the Molecular Genetics Laboratory of the Faculty of Medicine, Padjadjaran University, and Laboratory of the Department of Aerophysiology Lakespra dr.Saryanto TNI Air Force.

In this study were used 45 male Sprague-Dawley rats aged between 2 and 3 months weighing 200 to 400 g, obtained from the Animal Breeding Laboratory of PT.Biomedical Technology Indonesia, Bogor, West Java, Indonesia. One week before the hypoxia experiment, rats were placed in a laboratory for the animal to adapt in an air-conditioned room (22 ± 3°C) with light cycle lighting (06.00–18.00). Rats were well cared for and given mineral water and food ad libitum every day. Prior to the start of the study, the experimental design in this study received ethical approval from the Research Ethics Commission of Universitas Padjadjaran, Bandung, Indonesia: 419/UN6.KEP/EC/2021.

Sample size in this study was calculated using the Federer formula as follows:

( *n* – 1) ( *k* – 1) ≥ 15

where:

*n* : number of samples per group
*k* : number of groups

( *n* – 1) (9–1) ≥ 15

8 *n* – 8 ≥ 15

*n* ≥ 2.9

The minimum sample for this study, per group, is 2.9 rounded up to 3. There was anticipation of dropping out, so 3 becomes 5 ( *n* = 5), meaning that the number of rats in each group is 5, so the total number of Sprague-Dawley rats is 45 rats.

Forty-five male Sprague-Dawley rats were then randomly divided into nine groups: four IHH groups, four normoxia groups, and one control group. They are as follows:

- IHH groups consisting of animals were given HH exposure for 30 minutes every day in the Hypobaric Chamber, namely group P1 (one time HH exposure, terminated on day 1, *n* = 5), group P2 (3 times HH exposure, terminated on day 3, *n* = 5), group P3 (5 times HH exposure, terminated on day 5, *n* = 5), and group P4 (7 times HH exposure, terminated on day 7, *n* = 5).
- Normoxia groups consisting of animals were kept under normoxia condition and placed in the room with the same sea level, namely group K1 (terminated on day 1, *n* = 5), group K2 (terminated on day 3, *n* = 5), group K3 (terminated on day 5, *n* = 5), and group K4 (terminated on day 7, *n* = 5).
- Control group consisting of animals was kept under normoxia condition and terminated immediately after tooth extraction, namely group K0 (terminated on day 0, *n* = 5).

From all experimental animals in this study before being given treatment, the maxillary left first molar was extracted. Experimental rats before tooth extraction were anesthetized according to body weight (BW) and performed intraperitoneally using ketamine combined with xylazine in the amount of 0.1 mL/10 g BW.

The dose was prepared by mixing 1.0 mL of 100 mg/mL ketamine with 0.5 mL of 20 mg/mL xylazine. The volume of the ketamine and xylazine mixture present was added to the saline solution until a total of 10 mL was reached. A total of 10 mL of the combination of ketamine and xylazine was used as much as 0.1 mL/10 g BW. 19 After the anesthetic stage was reached, when the animals were in a deep sleep condition, tooth extraction was started.

Immediately after being anesthetized, teeth of the experimental rats were extracted using modified tooth extraction tools, namely a sterile dental periodontal surgical instrument and arterial clamps as special pulling pliers; with unidirectional movement, teeth were carefully pulled to avoid tooth fracture and the teeth were completely extracted. Then post-tooth extraction socket is cleaned with sterile gauze and allowed to undergo natural healing, and no suturing of the wound is performed on the tissue.

**Fig. 1:**
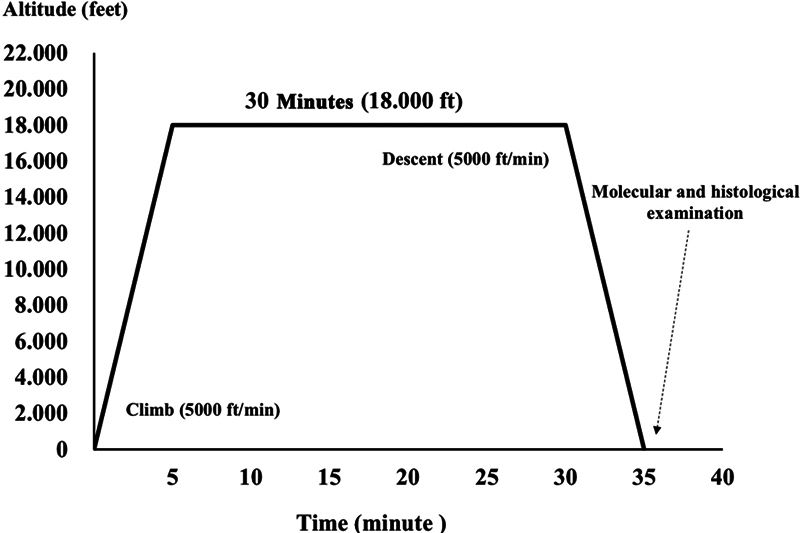
Altitude simulation of hypobaric hypoxia exposure procedure using the hypobaric chamber. Hypobaric hypoxia exposure in each procedure occurred at an altitude of 18,000 feet for 30 minutes.

HH exposure in the IHH group (groups P1, P2, P3, and P4) was performed by placing experimental rats into the Hypobaric Chamber for 30 minutes at an altitude of 18,000 feet and the air temperature was maintained at around 28°C with humidity around 58%. HH exposure was repeated every day for intermittent hypobaric hypoxic exposure, namely one time HH, three times HH, five times HH, and seven times HH. The HH procedure was designed based on special training for Indonesian Air Force Soldiers. 20 The hypobaric hypoxic procedure in this experiment is shown in Fig. 1 .

The experimental rats were terminated for socket tissue sampling performed on days 0, 1, 3, 5, and 7 after tooth extraction. The IHH group was terminated immediately at the end of each experiment after HH exposure at the ground level, the normoxic group was terminated at the end of each experiment, and the control group was terminated immediately on the day after tooth extraction.

The experimental animals before termination were anesthetized intraperitoneally using a combination of ketamine and xylazine in the amount of 0.1 mL/10 g BW. 19 After the anesthetic stage was reached, and the experimental animals were deep asleep, termination began.

Sampling from the socket tissue is performed when it is confirmed that the experimental animal is dead. The upper jaw was cut with a scalpel, and the post-extraction socket tissue was carefully extracted and divided into half with the thinnest separating disk drill. Half-socket tissue per animal of approximately 20 to 30 mg was rapidly placed in microtube centrifuge tubes, preserved with RNA later, and stored at −80°C until use for real-time polymerase chain reaction (RT-PCR), and the remaining tissue was fixed in formalin and processed for histological analysis.

### Measurement of mRNA Expression

Measurement of HIF-1α and VEGF messenger RNA (mRNA) expression was done using one-step RT-PCR. Samples were extracted using the RNeasy Mini Kit reagent from Qiagen. RT-PCR was performed using the Rotor-Gene Q Software 2.3.1.49 real-time PCR system from Qiagen with the SensiFAST SYBR No-ROX one-Step Kit for HIF-1α and VEGF. For mixing the material into a PCR tube, use SensiFAST SYBR (2X) 10 µL composition, Primer F 0.8 µL, Primer R 0.8 µL, Reverse Transcriptase Enzyme 0.2 µL, Ribosafe RNAse Inhibitor 0.4 µL, Nuclease Free Water 5.8 µL, and RNA Extract 2 µL. Insert the PCR tube and then set the RT-PCR cycle starting with the first incubation at 45°C for 10 minutes, followed by a second incubation at 95°C for 2 minutes, then denaturation 40 cycles at 95°C for 5 seconds, and extension at 60°C for 20 seconds. Instructions per manufacturer's protocol must be strictly followed.

The primary sequences are as follows:

HIF-1α: 5′-CTTTCTCTGCGCGTGAGGAC-3′ as forward, and 5′-TTCGACGTTCGGAACTCATCCT-3′ as reverse, and produce the amplicon size of the PCR product 149 bp.
VEGF-a: 5′-CTGGACCCTGGCTTTACTGC-3′ as forward, and 5′-AATTGGACGGCAATAGCTGCG-3′ as reverse, and produce the amplicon size of the PCR product 136 bp
β-actin: 5′-CACCCGCGAGTACAACCTTC-3′ as forward, and 5′-CCCATACCCACCATCACACC-3′ as reverse.

The mRNA expression was calculated using the Livak 2(−ΔΔCT) formula, by comparing the Ct values of the treatment group with the control group. 21 Gene expression value ≥ 1 indicates that gene expression has increased compared with control. β-actin is used as internal control. ΔCT = Ct target gene – Ct housekeeping gene. ΔΔCT = ΔCT treatment – ΔCT control. Gene expression = 2(−ΔΔCT).

### Histological Assay

Post-tooth extraction socket tissues were fixed with formalin combined with phosphate buffer saline-formalin solution for 24 hours at 4°C. Then the samples were decalcified with 10% ethylenediaminetetraacetic acid (EDTA) solution at pH 7.4 and stored at temperature of 4°C for 6 to 8 weeks, depending on the degree of mineralization with EDTA, and renewed every 3 days. Then they were trimmed and arranged into tissue cassettes and labeled; the dehydration stage is performed next by immersing the tissues into an alcohol solution in stages starting from 70%, then 80%, 90%, 95%, and finally 100%. They are cleaned three times with xylene solution for 60 minutes each per cycle, then infiltrated in liquid paraffin in three cycles by immersing the tissue into liquid paraffin for 60 minutes each per cycle, blocking until the paraffin freezes. The tissues were cut using a microtome slicer that were then made into slide preparations and stained with hematoxylin and eosin (H&E); then the preparations were glued using entellan and covered with a cover glass. The dry slides were observed under a binocular lens microscope (Olympus Type CX31) ×400 magnification equipped with a camera with five different fields of view. The photos produced by the camera were transferred to a computer and evaluated with the Tool Image J software. Histological analysis of expressed angiogenesis was quantified by counting neovascular cells with new vessel images, then tabulated and data analyzed.

### Statistical Analysis

Statistical tests analyzed the data of this research using the MegaStat V.10.4 release 3.2.4 Mac software. The data normality test used the chi-square test ( *p* > 0.01), and the result was that all data were normally distributed. Meanwhile, the variant homogeneity test used the Bartlett test, and the result was that all data had a homogeneous variant. The data results are presented as mean ± standard deviation (SD). Data were analyzed using the one-way analysis of variance (ANOVA) test followed by post hoc *t* -test analysis to analyze the differences between the experimental group and the control group. The difference was statistically significant when it showed a value of *p* less than 0.05. When the data results do not met the parametric test, then a nonparametric test was performed with the Kruskal–Wallis test followed by Mann–Whitney U test analysis to analyze differences in expression between groups.

## Results

All experimental animals ( *n* = 45) were in a safe condition and no complications occurred after tooth extraction. The findings of each group were evaluated and calculated on days 0, 1, 3, 5, and 7 after tooth extraction. HIF-1α mRNA, VEGF mRNA, and angiogenesis expressions were measured and compared between the experimental group (IHH and normoxia) and the control group. HIF-1α was the main regulatory molecule for oxygen homeostasis under hypoxic condition and determined the outcome of wound healing through the activation of several target genes. HIF-1α expression was detected in all IHH and normoxia groups and the control group. The results of the one-way ANOVA test found a significant difference, *p* = 0.0019 (** *p* < 0.05), indicating that there was an effect of IHH exposure on changes in HIF-1α mRNA expression in the socket after tooth extraction; then a post hoc *t* -test was performed to examine the differences between each group. Post hoc *t* -test results found differences in HIF-1α mRNA expression after HH exposure in the IHH group (groups P1, P2, P3, and P4), and the normoxia group (groups K1, K2, K3, and K4) compared with the control group (K0). The results of data analysis are shown as mean ± SD in Table 1 .

**Table 1:** Differences of HIF-1α mRNA expression after IHH exposure, normoxia, and control

HIF-1α	Day 0	Day 1	Day 3	Day 5	Day 7
Control	1.0				
IHH		3.136 ± 1.069*	2.509 ± 0.805 a	2.073 ± 0.904	1.114 ± 0.560
Normoxia		2.165 ± 1.669	1.039 ± 0.534	0.751 ± 0.777	0.413 ± 0.300

Abbreviations: ANOVA, analysis of variance; HIF-1α, hypoxia-induced factor-1α; IHH, intermittent hypobaric hypoxia; mRNA, messenger ribonucleic acid; SD, standard deviation.

Note: Values presented are mean ± SD.

a *p* < 0.05, one-way ANOVA test; significantly different compared with the control group.

In the IHH group, HIF-1α mRNA expression was found after 1 time HH exposure on day 1 in the P1 group (3.136 ± 1.069) in which there was a significant increase, *p* = 0.0033 (** *p* < 0.05), compared with the control group, and after 3 times HH exposure on day 3 in the P2 group (2.509 ± 0.805), there was a decrease compared with the P1 group but still it increased significantly, *p* = 0.0323 (* *p* < 0.05), above the control group; then after 5 times HH exposure on day 5 in the P3 group (2.073 ± 0.904), there was a decrease approaching the control group with a value of *p* = 0.1221 and, after 7 times HH exposure on day 7 in the P4 group (1.114 ± 0.560), there was a further decrease but still above the control group with a value of *p* = 0.8673, indicating that the P3 and P4 groups were not significantly different from the control group.

**Fig. 2:**
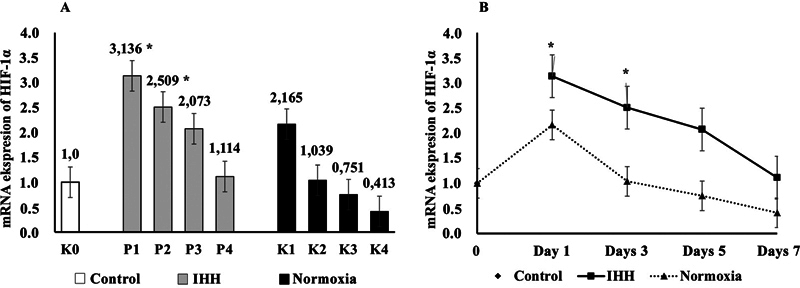
Hypoxia-induced factor-1α (HIF-1α) messenger ribonucleic acid (mRNA) expression in post-tooth extraction socket after intermittent hypobaric hypoxia exposure. ( **A)** Group K0 (control on day 0); hypobaric hypoxic groups, namely group P1 (one time hypobaric hypoxia [HH] exposure on day 1), group P2 (three times HH exposure on day 3), group P3 (five times HH exposure on day 5), and group P4 (seven times HH exposure on day 7); and the normoxia groups, namely group K1 (normoxia on day 1), group K2 (normoxia on day 3), group K3 (normoxia on day 5), and group K4 (normoxia on day 7). ( **B)** HIF-1α mRNA expression in the socket after tooth extraction after intermittent hypobaric hypoxia exposure in normoxia group on days 1, 3, 5, and 7. It was significantly different compared with the control group (* *p* < 0.05, one-way analysis of variance test). IHH, intermittent hypobaric hypoxia.

In the normoxia group, HIF-1α mRNA expression was found in the K1 group (2.165 ± 1.669) on day 1, in which there was an increase with a value of *p* = 0.0944, and in group K2 (1.039 ± 0.534) on day 3, there was a decrease but still it was above the control group with a value of *p* = 0.9542; then in the K3 group (0.751 ± 0.777) on day 5, there was a decrease below the control with a value of *p* = 0.7151, and in group K4 (0.413 ± 0.300) on day 7, it increasingly decreased under the control group with a value of *p* = 0.2534, indicating that the K1, K2, K3, and K4 groups were not significantly different from the control group. The results of the analysis of differences in HIF-1α mRNA expression are shown in Fig. 2 .

VEGF after hypobaric hypoxic exposure was also measured in this study. VEGF as a growth factor is a target gene for HIF-1α in a hypoxic condition, playing a role in facilitating tissue repair by increasing vascular permeability that promotes the migration of inflammatory cells to the wound site, stimulates angiogenesis, and grows new blood vessels that promote wound healing. VEGF-a mRNA expression was detected in all IHH and normoxia groups and the control group on days 0, 1, 3, 5, and 7 after tooth extraction. The results of the one-way ANOVA test found a significant difference, *p* = 0.0000000064 (** *p* < 0.05), indicating that there was an effect of IHH exposure on changes in VEGF-a mRNA expression in the socket after tooth extraction. Then a post hoc *t* -test was performed to test whether there was a difference between each group. Post hoc *t* -test results found that there were differences in VEGF-a mRNA expression after exposure to HH in the IHH group (groups P1, P2, P3, and P4) and the normoxia group (groups K1, K2, K3, and K4) compared with the control group (group K0). The results of data analysis are shown as mean ± SD in Table 2 .

**Table 2:** Differences of VEGF mRNA expression after IHH exposure, normoxia, and control

VEGF	Day 0	Day 1	Day 3	Day 5	Day 7
Control	1.0				
IHH		1.822 ± 0.315	1.957 ± 0.619	3.547 ± 0.243 a	4.153 ± 0.432 a
Normoxia		0.712 ± 0.378	0.888 ± 0.539	1.263 ± 0.162	3.351 ± 0.612 a

Abbreviations: ANOVA, analysis of variance; IHH, intermittent hypobaric hypoxia; mRNA, messenger ribonucleic acid; SD, standard deviation; VEGF, vascular endothelial growth factor.

Note: Values presented are mean ± SD.

a *p* < 0.05, one-way ANOVA test; significantly different compared with the control group.

In the IHH group, VEGF-a mRNA expression was found after 1 time HH exposure on day 1 in the P1 group (1.822 ± 0.315), which increased compared with the control group with a value of *p* = 0.1023, and after 3 times HH exposures on day 3 in the P2 group (1.957 ± 0.619), there was an increase with a value of *p* = 0.0586, indicating that the P1 and P2 groups were not significantly different from the control group; then after 5 times HH exposure on day 5 in the P3 group (3.547 ± 0.243), there was a significant increase, *p* = 0.000008 (** *p* < 0.05), and after 7 times HH exposure on day 7 in the P4 group (4.153 ± 0.432), a significant increase, *p* = 0.0000002 (** *p* < 0.05), was observed compared with the control group.

**Fig. 3:**
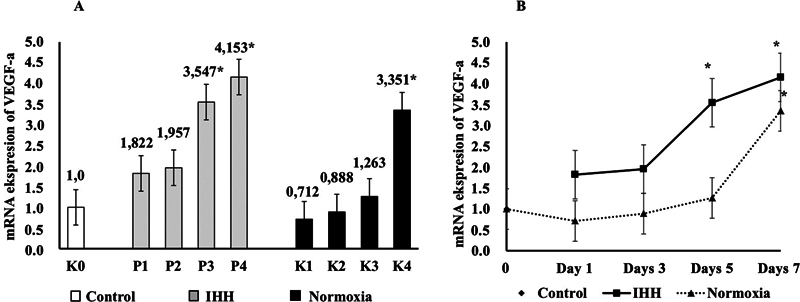
Vascular endothelial growth factor-a (VEGF-a) messenger ribonucleic acid (mRNA) expression in post-tooth extraction socket after intermittent hypobaric hypoxia exposure. ( **A)** Group K0 (control on day 0); hypobaric hypoxic groups, namely group P1 (one time hypobaric hypoxia [HH] exposure on day 1), group P2 (three times HH exposure on day 3), group P3 (five times HH exposure on day 5), and group P4 (seven times HH exposure on day 7); and the normoxia groups, namely group K1 (normoxia on day 1), group K2 (normoxia on day 3), group K3 (normoxia on day 5), and group K4 (normoxia on day 7). ( **B)** VEGF-a mRNA expression in the socket after tooth extraction after intermittent hypobaric hypoxia exposure in normoxia group on days 1, 3, 5, and 7. Significantly different compared with the control group (* *p* < 0.05, one-way analysis of variance test). IHH, intermittent hypobaric hypoxia.

In the normoxia group, there was a change in VEGF-a mRNA expression in group K1 (0.712 ± 0.378) on day 1 with a value of *p* = 0.5603 but still it was below the control group, and group K2 (0.888 ± 0.539) on day 3 with a value of *p* = 0.8206 also was still below the control group, but group K3 (1.263 ± 0.162) on day 5 began to increase above the control group with a value of *p* = 0.5942, indicating that groups K1, K2, and K3 were not significantly different from the control group, while the K4 group (3.351 ± 0.612) on day 7 experienced a significant increase, *p* = 0.00003 (** *p* < 0.05), compared with the control group. The results of the analysis of differences in HIF-1α mRNA expression are shown in Fig. 3.

The amount of angiogenesis in post-tooth extraction socket after exposure to HH was also measured on days 0, 1, 3, 5, and 7. Angiogenesis is the formation of new blood vessels through the growth of capillary branches from existing vascular tissue, which occurs under the activity of synergistic factor VEGF growth that plays an important role in the wound-healing process. Angiogenesis was detected in all IHH and normoxia experimental groups and the control group. The results of the Kruskal–Wallis test found that there was a significant difference, *p* = 0.00044 (*** *p* < 0.05), indicating that there was an effect of IHH exposure on changes in the amount of angiogenesis in the post-tooth extraction socket; then the Mann-Whitney test was performed to test whether there were differences between each experimental group. The results of the Mann-Whitney test showed that there was a difference in the amount of angiogenesis after exposure to HH in the IHH group (groups P1, P2, P3, and P4), and the normoxic group (groups K1, K2, K3, and K4) compared with the control group (K0).

This study found a change in the amount of angiogenesis in the IHH group. After 1 time HH exposure on day 1 in the P1 group (11.00), there was a significant increase, *p* = 0.0049 (** *p* < 0.05), compared with the control group; then after 3 times HH exposure on day 3 in group P2 (29.80), there was a very significant increase, *p* = 0.000000003 (** *p* < 0.05), and after 5 times HH exposure on day 5 in the P3 group (32.70), it had a significant increase, *p* = 0.0000000005 (** *p* < 0.05); then after 7 times HH exposure on day 7 in the P4 group (35.60), there was a significant increase, *p* = 0.0000000001 (** *p* < 0.05), compared with the control group, which showed that all IHH groups (P1, P2, P3, and P4) experienced an increase compared with the control group.

**Fig. 4:**
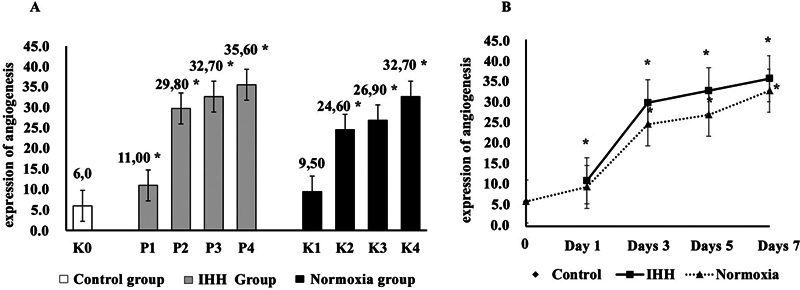
Total angiogenesis in post-tooth extraction socket after intermittent hypobaric hypoxia exposure. ( **A)** Group K0 (control on day 0); hypobaric hypoxic groups, namely group P1 (one time hypobaric hypoxia [HH] exposure on day 1), group P2 (three times HH exposure on day 3), group P3 (five times HH exposure on day 5), and group P4 (seven times HH exposure on day 7); and the normoxia groups, namely group K1 (normoxia on day 1), group K2 (normoxia on day 3), group K3 (normoxia on day 5), and group K4 (normoxia on day 7). ( **B)** Total angiogenesis in the socket after tooth extraction after intermittent hypobaric hypoxia (IHH) exposure in normoxia group on days 1, 3, 5, and 7. Significantly different compared with the control group (* *p* < 0.05, Kruskal–Wallis test).

In the normoxia group, a change in the amount of angiogenesis was found. In the K1 group (9.50) on day 1, there was a very significant increase, *p* = 0.0217 (** *p* < 0.05), compared with the control group; then in the K2 group (24.60) on day 3, there was a very significant increase, *p* = 0.0000000001 (** *p* < 0.05), and in the K3 group (26.90) on day 5 there was a very significant increase, *p* = 0.00000002 (** *p* < 0.05); then in the K4 group (32.70) on day 7, there was a significant increase, *p* = 0.0000000005 (** *p* < 0.05), compared with the control group, indicating that all normoxia groups (K1, K2, K3, and K4) increased compared with the control group. The results of the analysis of differences in the number of angiogenesis are shown in Fig. 4.

**Fig. 5:**
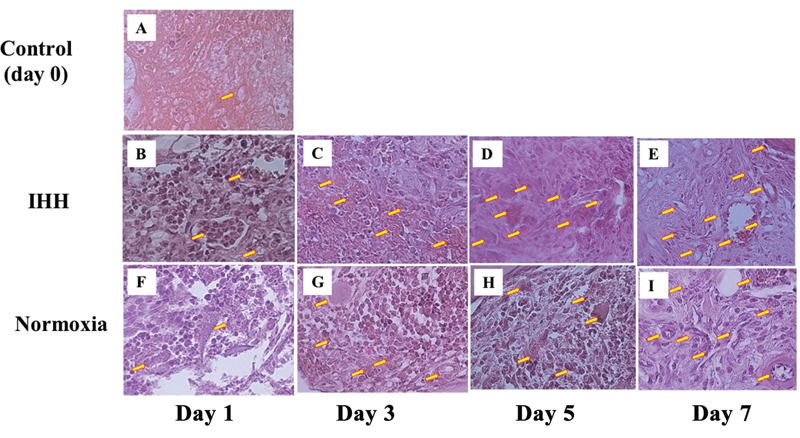
Histological test of angiogenesis in post-tooth extraction socket after intermittent hypobaric hypoxia exposure. **(A)** Control group (normoxia on day 0). Intermittent hypobaric hypoxia (IHH) groups, namely: **(B)** one-time hypobaric hypoxia [HH] exposure group on day 1, **(C)** three times HH exposure group on day 3, **(D)** five times HH exposure group on day 5, and **(E)** seven times HH exposure group on day 7. Normoxia groups, namely: **(F)** normoxia group on day 1, **(G)** normoxia group on day 3, **(H)** normoxia group on day 5, and **(I)** normoxia group day 7. Magnification ×400; yellow arrow indicates angiogenesis; hematoxylin and eosin staining.

In this study, the histological results of angiogenesis were found in the post-tooth extraction socket in the IHH group, the normoxia group, and the control group on days 0, 1, 3, 5, and 7 with H&E staining; angiogenesis had begun to appear on day 1, and increased on day 3, then increased on day 5, and reached the highest peak on day 7 after tooth extraction. Histological picture of angiogenesis is shown in Fig. 5 .

## Discussion

In this study, we investigated the direct impact of IHH exposure on rats after tooth extraction. After being exposed to HH in the Hypobaric Chamber, by simulating flying for 30 minutes every day at an altitude of 18,000 feet, with exposure of 1 time HH, 3 times HH, 5 times HH, and 7 times HH, molecular changes were found in the socket after tooth extraction in hypoxic conditions as a regulator of wound healing, describing that flying at high altitudes will result in HH that can activate several target genes in response to hypoxia. Early wounds will cause hypoxia due to disruption of blood vessels that activate a series of molecular events needed in the wound-healing process. Tissue hypoxia induces a sustained increase in HIF-1α expression as a major regulator of oxygen homeostasis and transcriptionally regulates the expression of many other genes that enhance the wound-healing process. 7

The result of this study found that HIF-1α mRNA expression in the post-tooth extraction socket increased significantly (** *p* < 0.05), and reached the highest after one time HH exposure on day 1, illustrating that acute HH condition with low-level oxygen increased HIF-1α expression even higher. HIF-1α mRNA expression began to decrease after three times HH on day 3, and further decreased after five times HH exposure on day 5, and continued to decrease closer to the control after seven times HH exposure on day 7. The result of this study described that there was an increase in HIF-1α mRNA expression in the socket after tooth extraction under acute HH condition and there was a gradual systemic adaptation at the cellular and tissue level of socket wound after tooth extraction under intermittent hypobaric hypoxic condition. Previous research stated that after being given IHH exposure to experimental rat liver tissue, there was an increase in HIF-1α after one time HH and two times HH compared with the control group; then after three times HH and four times HH exposure, it would not increase the expression of HIF-1α, because there had been a gradual adaptation of hepatocytes to hypoxia. 18 HIF-1α expression was significantly increased under IHH exposure condition and returned to normal levels after repeated or intermittent hypobaric hypoxic exposure induced a protective response causing cells to adapt to intermittent hypobaric hypoxic conditions. 16 17 18 22 Changes in oxygen concentration due to hypoxia would modulate cell function by stabilizing HIF-1α, which was a transcription factor for many genes that regulated adaptive responses to hypoxia. 7 23 Stabilization of HIF-1α as the main regulator of oxygen homeostasis and a determinant of wound healing results through the activation of several HIF-1α target genes. 7

HIF-1α mRNA expression was also found in normoxia without HH exposure on days 1, 3, 5, and 7 after tooth extraction, indicating a difference compared with the control group. HIF-1α mRNA expression began to increase to the highest on day 1 after tooth extraction compared with the control group, illustrating initial wound hypoxia due to decreased oxygen levels below physiological levels, which modulated cell function by increasing HIF-1α. In the initial wound, there was disruption of blood vessels, which caused disruption of oxygen delivery to the wound site resulting in tissue hypoxia, which could induce increased expression of HIF-1α. 7 HIF-1α appeared as the most active isoform during short periods (2–24 hours) of hypoxia in some cell line. 24 HIF-1α mRNA expression on day 3 after tooth extraction began to decrease toward the control group, then on days 5 and 7 there was a further decline until it was below the control group, illustrating that oxygen supply during normoxic conditions returned to normal and supplied all the cells in the wound tissue. HIF-1α expression on cells under normoxia conditions degraded quickly and continuously, resulting in a decrease in HIF-1α transcriptional activity. 7 23 In normoxic conditions, when there is sufficient amount of oxygen, HIF-1a will be degraded, whereas under hypoxic conditions, HIF-1a undergoes stabilization by forming heterodimers with HIF-1β and then translocating to the nucleus and binding to Hypoxia-Response Element (HRE) elements promoting expression of target genes. 7 23 24 25

In this study, VEGF-a mRNA expression in the IHH group was higher than that in the normoxia group and the control group. The IHH group found that VEGF-a mRNA expression increased in all groups. VEGF-a mRNA expression in the IHH group began to increase after one time HH exposure on day 1 above the control group, then increased again after three times HH exposure on day 3, then increased further again after five times HH exposure on day 5, and then the highest increase occurred after seven times HH exposure on day 7, illustrating that relatively low oxygen conditions would respond to HIF-1α activity that could affect VEGF-a mRNA expression under HH condition that is regulated by the HIF-1α transcription factor. The effect of IHH on VEGF expression was highest on day 7, indicating that the formation of new blood vessels had started to support the healing process better. VEGF could promote endothelial cell proliferation, migration, and angiogenesis by promoting mitochondrial function. 26 Increased accumulation of VEGF via its receptor (VEGFR) on endothelial cells induced angiogenesis and indirectly increased the oxygen supply that is required for angiogenesis. Overall, the HIF-1α-VEGF pathway linked the process of angiogenesis. 27 Activation of HIF-1α could activate VEGF expression, and played an important role in angiogenesis and stimulated the formation of new blood vessels at tooth extraction sites that affected wound healing. 7 8 Regulation of angiogenesis by HIF-1α is very important for stimulating the formation of new blood vessels, increasing blood supply, returning oxygen, and delivery of nutrients to the wound site, thus increasing cell survival, which promotes wound healing. 28

VEGF-a mRNA expression during normoxia condition without HH exposure on days 1, 3, 5, and 7 after tooth extraction in this study did not experience much change. VEGF-a mRNA expression in the normoxia group on days 1 and 3 after tooth extraction had not increased and was still below the control group, whereas on day 5, it began to increase, and the highest increase occurred on day 7 after tooth extraction, which describes the normoxic condition in line with the normal wound healing. Another study was reported on normal wound healing, which showed that VEGF increased on days 3 to 5 and would decrease from days 7 to 14; after tooth extraction injury, VEGF level and activity decreased and a decrease in granulation tissue formation was observed. 29 VEGF and angiogenesis expression reached a peak in the first week after surgery, and then levels progressively decreased until the fourth week of healing. 30 The normoxia situation was different when compared with after of IHH exposure. The increase in VEGF-a mRNA expression occurred faster and was higher in the IHH group compared with the normoxia group and the control group, illustrating that at the beginning of the wound, tissue hypoxia occurred and the provision of IHH exposure with low oxygen levels caused HIF-1α to be activated, resulting in an increase in the amount of VEGF-a mRNA expression, which was higher than the normoxia group and the control group.

The result of histological tests in this study found that the amount of angiogenesis in the post-tooth extraction socket after IHH exposures in the normoxia group began to increase on days 1, 3, and 5, and reached the highest peak on day 7. The increase in the number of angiogenesis occurred faster and higher in the IHH group compared with the normoxia group and the control group, illustrating that at the beginning of the wound, tissue hypoxia occurred plus the provision of IHH exposure with low oxygen levels, activating the VEGF growth factor, resulting in an increase in the amount of angiogenesis, which was higher than the normoxia group and the control group. This increase in the number of angiogenesis was in line with the increase in VEGF-a mRNA expression, illustrating that VEGF-a mRNA expression under hypobaric hypoxic condition could be regulated by the transcription factor HIF-1α. VEGF significantly promoted proliferation, migration, angiogenesis, and the cell cycle of endothelial cells. 26 The relatively low O _2_ environment at the start of injury causes hypoxic tissue to express HIF-1α, which activates proangiogenic transcription factors, such as VEGF, as the main regulators of vascular growth stimulus angiogenesis. 8 31 Angiogenesis is the formation of new blood vessels through the growth of new capillary branches from the existing vascular tissue and occurs under the synergistic activity of the VEGF growth factor, which is important in accelerating tissue repair in the wound-healing process. 28

The limitation of this study is that this is a preliminary study to investigate the effect of IHH exposure on changes in HIF-1α mRNA, VEGF mRNA, and angiogenesis expression after tooth extraction in rats as the model, and this study was only performed for 7 days with seven times HH exposure and the clinical examination analysis of the post-tooth extraction wound was not recorded.

## Conclusion

In conclusion, the present study ascertained that IHH exposure increases the amount of angiogenesis in the post-tooth extraction socket. The increased HIF-1α mRNA and VEGF-a mRNA expressions can stimulate the formation of new blood vessels, increase blood supply, and accelerate wound healing, thus becoming the basis for targeted and appropriate post-tooth extraction wound-healing therapy in the future according to the patient's adaptation to the altitude. Further research is needed to evaluate the effect of IHH for more than 7 days with exposure to more than seven times HH and radiographic examination.
